# Fixed Gonadotropin-Releasing Hormone Antagonist Protocol *Versus* Flexible Progestin-Primed Ovarian Stimulation Protocol in Patients With Asynchronous Follicular Development During Controlled Ovulation Stimulation: A Retrospective Study

**DOI:** 10.3389/fendo.2021.690575

**Published:** 2021-11-18

**Authors:** Mei Dong, Li Sun, Li Huang, Fang Wang, Xiqian Zhang, Fenghua Liu

**Affiliations:** Department of Reproductive Medical Center, Guangdong Women and Children Hospital, Guangzhou, China

**Keywords:** fixed gonadotropin-releasing hormone antagonist protocol, flexible progestin-primed ovarian stimulation protocol, asynchronous follicular development, controlled ovulation stimulation, gonadotropin releasing hormone, medroxyprogesterone acetate

## Abstract

Protocols utilizing gonadotropin-releasing hormone (GnRH) antagonists have emerged as mainstream procedures for ovarian stimulation; however, GnRH increases the risk for periodic cancellation of embryos. Therefore, this study aimed to compare the pregnancy outcomes of a fixed GnRH antagonist protocol and a flexible progestin-primed ovarian stimulation (fPPOS) protocol in patients with asynchronous follicular development during controlled ovulation stimulation and to explore the feasibility of converting patients undergoing a fixed GnRH antagonist protocol to an fPPOS protocol. This was the first retrospective study exploring the fPPOS protocol in patients with asynchronous follicular development, and it was conducted in a public reproductive medicine center from January to December 2020. We included infertile women. All participants were scheduled to undergo administration of a GnRH antagonist on the fifth day of controlled ovulation stimulation. The study group included 129 women who were converted from the fixed GnRH antagonist protocol to the fPPOS protocol for their asynchronous follicular development, while the antagonist group consisted of 258 women (ratio 1:2) who proceeded with a fixed GnRH antagonist protocol. On the second or third day of the menstrual period, 100–300 IU/day gonadotropin injections were administered. For patients who were converted to the fPPOS protocol, medroxyprogesterone acetate tablets at 10 mg/day were started on the fifth day of stimulation or when only one leading follicle reached 14 mm and the other follicles were ≤10 mm in diameter, whichever came first. The rates of embryo implantation, clinical pregnancy, and early pregnancy loss were obtained. The number of oocytes retrieved and the number of high-quality embryos in the antagonist group were significantly higher than those in the fPPOS group (*P* = 0.039 and *P* = 0.025, respectively). No significant differences in the rates of embryo implantation, clinical pregnancy, and early pregnancy loss were observed between the two groups. Our study found that in patients who were scheduled for administration of GnRH antagonists but presented with asynchronous follicular development on the fifth stimulation day, it was feasible to switch to the fPPOS protocol.

## Introduction

Gonadotropin-releasing hormone (GnRH) antagonists were developed approximately 40 years ago. However, only recently has their use become widespread in clinical practice (1). GnRH antagonists inhibit the secretion of the pituitary luteinizing hormone (LH), which, in turn, inhibits premature follicular ovulation (2, 3). Protocols utilizing GnRH antagonists have emerged as mainstream procedures employed in ovarian stimulation because they do not require pituitary downregulation, and they utilize a low gonadotropin (Gn) dosage and have short treatment cycles and good patient compliance (4, 5). Compared with the shorter learning curve period of the GnRH agonist protocol, that of the antagonist protocol was relatively longer. Numerous studies regarding antagonist protocols focused on the population to whom these protocols could be applied, the success rate of fresh embryo transfer, and the influence of hormone levels on pregnancy outcomes (6–8). However, only few studies have explored the flexible conversion of antagonist protocols. Furthermore, the original antagonist protocols were unsuitable for implementation.

Since each follicle in a follicular cluster requires different thresholds of follicle-stimulating hormone (FSH), the development of follicles is not uniform and synchronized (9). Several studies reported that administration of exogenous Gn during the follicular development cycle artificially extended the FSH window period and changed the physiological mechanism of a single dominant follicle maturation under certain conditions (9, 10). Furthermore, the administration of exogenous Gn at the end of the FSH window rescued the follicles that would have been locked, thereby recruiting more follicles to continue to grow (11). However, owing to the different threshold and sensitivity of follicles, the developmental asynchrony was further expanded (10, 12). In one study, non-uniformity, also known as non-synchrony, was defined as a 3- to 4-mm difference in the diameters of the dominant and secondary follicles in the process of controlled ovulation induction (12).

Since the introduction of GnRH antagonists, numerous studies have attempted to determine the optimal timing of its administration (13). The first GnRH antagonist protocol was a fixed protocol, which was based on the administration of GnRH antagonists on the fifth or sixth day of the menstrual cycle (14). Subsequently, a flexible protocol was adopted to reduce the number of GnRH antagonist injections (15). Although preferences have been different, both protocols have been widely used (13). However, most research studies evaluating GnRH antagonists focused on the number of days of Gn stimulation or the diameter of the follicle and did not consider remedial options (16). However, in our clinical practice and other studies (17), some patients with controlled ovulation stimulation (COS) had uneven follicular growth after Gn administration. Moreover, routine administration of antagonists often resulted in an artificial low response to Gn (18). It also resulted in premature luteinization of follicles and ovulation (7). Therefore, there was a risk of periodic cancellation.

The progestin-primed ovarian stimulation (PPOS) protocol was proposed by the Yanping Kuang Medical Doctor Group in 2015 (19). From the early stage of the follicle, oral exogenous progesterones, such as medroxyprogesterone acetate (MPA), and dydrogesterone (20–23), were used together with Gn during COS (24). The PPOS protocol effectively prevented estradiol (E2)-induced LH activation and transmission phase. Thus, it can be used as an alternative to conventional treatment with GnRH analogs.

This was the first retrospective study that aimed to compare pregnancy outcomes of a fixed GnRH antagonist protocol and a flexible progestin-primed ovarian stimulation (fPPOS) protocol in patients with asynchronous follicular development during COS and to explore the feasibility of converting patients undergoing a fixed GnRH antagonist protocol to an fPPOS protocol.

## Materials and Methods

The protocol of this retrospective study was approved by the Ethics Committee of the Guangdong Women and Children Hospital.

### Study Population

Patients who underwent assisted reproductive technology treatment for infertility in our department from January 1, 2020, to December 31, 2020, were unselectively and consecutively screened according to the inclusion and exclusion criteria provided below. Only the first COS cycle was selected. The subsequent cycles were excluded from the cohort.

The inclusion criteria were as follows: 1) women under 36 years of age, 2) an anti-Mullerian hormone (AMH) level ≥1 ng/mL, 3) women undergoing their first *in vitro* fertilization/intracytoplasmic sperm injection/pre-implantation genetic testing (IVF/ICSI/PGT)-assisted reproduction, 4) women who started using Gn during the follicular phase, 5) women who had only one follicle with a diameter ≥14 mm, while that of the remaining follicles was ≤10 mm, 4 days after the initiation of Gn, and 6) women who implemented the freeze-all program.

The exclusion criteria were as follows: 1) polycystic ovary syndrome and 2) severe endometriosis (grade 3 or above).

### fPPOS Stimulation Protocols and Pituitary Suppression

**Figure 1:**
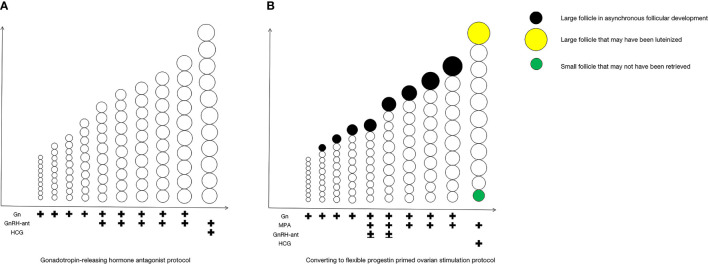
COS process of fixed GnRH antagonist protocol and fPPOS stimulation protocols with asynchronous follicular development. The COS process of the fixed GnRH antagonist protocol in **(A)**, and the COS process of the fPPOS stimulation protocols in **(B)**. COS, controlled ovulation stimulation; Gn, gonadotropin; GnRH-ant, gonadotropin-releasing hormone (GnRH) antagonists; HCG, human chorionic gonadotropin; MPA, medroxyprogesterone acetate.

For clarity, we show the COS process of the fPPOS stimulation protocols in **Figure 1B**. After a baseline scan on the second or third day of the menstrual cycle to exclude any follicles greater than 12 mm and any ovarian pathology that would debar ovarian stimulation, approximately 100 to 300 IU/day Gn injections were administered. The dosage of Gn depended on the patient’s age, body mass index (BMI), antral follicle count (AFC), and AMH levels. Stimulation was monitored using ultrasound and serum estradiol (E2), LH, and progesterone (P) levels every 2 to 4 days, as deemed necessary. Gn dosage was adjusted according to serum hormone levels and follicle measurements. MPA tablets with a dose of 10 mg/day were administered on the fifth day of stimulation or when only one dominant follicle reached 14 mm, while the other follicles had smaller diameters less than or equal to 10 mm, whichever came first. GnRH-ant was possibly administered on days 5 and 6 of the cycle in the fPPOS group. When three or more follicles reached a diameter of 17 mm or at least one dominant follicle reached a diameter of 18 mm, 10,000 units of human chorionic gonadotropin (HCG) were administered to induce final oocyte maturation. The final doses of MPA were administered on the day of HCG injection. Transvaginal oocyte retrieval under general anesthesia was performed 36 hours after the HCG injection. All patients underwent the freeze-all strategy.

### Matching Method of the Antagonist Group

R4.0.2 software was used for propensity score matching. The medication regimen was the grouping variable. The antagonist group was the antagonist group. The patients’ age, BMI, AFC, and AMH levels were included as covariates in the model. The matching algorithm selected the nearest-neighbor matching method. The matching ratio was set to 1:2, while the caliper value was set to 0.02.

### Control Stimulation Protocols and Pituitary Suppression in the Fixed GnRH Antagonist Protocol

For clarity, we show the COS process of the fixed GnRH antagonist protocol in **Figure 1A**. GnRH antagonist injections (0.25 mg) were administered on the fifth day of stimulation. Gn dosage adjustment, trigger timing, the trigger plan, and the egg retrieval time were the same as those in the fPPOS protocol. All patients in the matched antagonist group underwent a freeze-all strategy.

### Embryo Treatment

Good-quality embryos (Grade A: uniform or slightly uneven with <10% fragmentation; Grade B: uniform or non-uniform blastomere size with 10%–20% fragmentation) were frozen on the third day of stimulation. Embryos that did not have good quality for cryopreservation were placed in an extended culture until the blastocyst stage. At this stage, on the fifth or sixth day of stimulation, only blastocysts with good morphology were frozen. The embryo quality assessment on the fifth and sixth days was based on the scoring system of Gardner and Schoolcraft. Embryos with a grade of R3BB were considered good blastocysts.

### Frozen-Thawed Embryo Transfer Strategy

FET procedures for cleavage-stage embryos and blastocysts were used. Embryo vitrification was carried out using the Cyrotop carrier system, with dimethylsulfoxide-ethylene glycolsucrose as a cryoprotectant. Embryos were then transferred into a series of diluted sucrose solutions (1, 0.5, and 0 mol/L sucrose) for thawing. Serum β-HCG levels were measured 12 to 14 days after embryo transfer. Subsequent ultrasound examinations were performed at a gestational age of 10 weeks.

### Outcome Measures

The duration of stimulation, total Gn use, pituitary suppression on the first day of stimulation, duration of pituitary suppression, and the number of cumulus oocyte complexes and metaphase II oocytes were compared between the fPPOS and the GnRH antagonist protocol groups. The number of oocytes retrieved, allocated metaphase II oocytes, suboptimal oocyte yield (the ratio between the total number of oocytes retrieved and the number of follicles with a mean diameter >10 mm on the trigger day), maturity rate of suboptimal oocyte yield, two pronuclear fertilized oocytes, cleavage rate per metaphase II oocyte, blastulation rate per metaphase II oocyte, embryo and rates of embryo implantation, clinical pregnancy rate, and early abortion rate per FET were compared between the two groups. The embryo implantation rate was calculated as the number of embryos with cardiac activity divided by the number of transferred embryos.

One or more gestational sacs were observed *via* ultrasound to diagnose clinical pregnancy, including normal intrauterine pregnancy, ectopic pregnancy, and simultaneous intrauterine and extrauterine pregnancy. Only the gestational sac was seen without the fetal heart. Multiple gestational sacs were counted as clinical pregnancies. Clinical pregnancy rate per freeze-thaw transplant cycle was calculated as the number of clinical pregnancy cycles divided by the number of freeze-thaw transplant cycles multiplied by 100%.

After the confirmation of pregnancy, spontaneous abortion within 12 weeks of pregnancy was termed as an early abortion. Biochemical pregnancy was an exception to this. Early abortion rate was calculated as the number of spontaneous abortion cycles within 12 weeks of gestation divided by the number of clinical pregnancy cycles multiplied by 100%.

### Statistical Considerations

All data were analyzed using SPSS 23.0 software. We performed normality tests on all quantitative variables. Among them, variables that obey the normal distribution are described by the mean ± standard deviation (x ± s), and the means of the groups were compared with the two-sample independent t-test. In addition, we used the median (interquartile range) [M(P25-P75)] to describe variables that do not follow the normal distribution, and we used the Mann-Whitney U test for comparisons between groups. We used percentage (%) to express qualitative variables and used the chi-squared test for comparisons between groups. α=0.05 is the test level, P<0.05 indicates a statistically significant difference.

## Results

### Baseline Characteristics of the Study Population

In accordance with our inclusion and exclusion criteria, we included 129 patients who had inconsistent follicle growth during controlled ovulation stimulation. We then performed the fPPOS protocol. According to the matching criteria of our experiment (age, BMI, AFC, and AMH), we included 258 patients (ratio 1:2) in the antagonist COS protocol, which was the antagonist group. As shown in **Table 1**, there were no significant differences in age, years of infertility, BMI, AFC, AMH, primary infertility ratio, or infertility factors between the fPPOS and antagonist groups, which also verified the effectiveness of the matching software. The basal hormone level of E2 and FSH were equal between the two groups, but the basal LH hormone level was higher in the fPPOS group than in the antagonist group.

**Table 1:** Baseline characteristics of the study population.

GROUP	fPPOS (n=129)	GnRH antagonist (n=258)	t/Z/chi-square value	P
Age (years)	34.56 ± 5.36	34.28 ± 5.23	0.484	0.629
Years of Infertility	3 (1-6)	3 (1-5)	-0.244	0.807
BMI (kg/cm2)	22.79 ± 2.99	22.64 ± 2.93	0.479	0.632
AFC	7 (4-14)	8 (5-14)	-0.736	0.461
AMH (ng/ml)	1.93 (0.7-4.84)	2.01 (1.03-4.93)	-0.941	0.347
Primary Infertility ratio	34.88% (45/129)	34.11% (88/258)	0.711	0.399
Baseline E2	39.14 (29.69-53.37)	37.42 (28.44-49.21)	0.620	0.535
Baseline FSH	7.50 (6.38-9.26)	7.37 (5.95-8.81)	1.631	0.103
Baseline LH	5.41 (3.74-7.64)	4.76 (3.52-6.30)	2.344	0.019
Type of Infertility			5.0657	0.167
Fallopian Tube and Pelvic Factors	31.01% (40/129)	39.53% (102/258)		
Ovulation Disorders and Endometriosis	6.98% (9/129)	4.27% (11/258)		
Male Factor	12.40% (16/129)	15.50% (40/258)		
Other Factors and PGT	49.61% (64/129)	40.70% (105/258)		

BMI, body mass index; AFC, antral follicular count; AMH, Anti Mullerian hormone. Statistical significance was reached at P < 0.05.

### Data on the Process of Ovulation Induction

As shown in **Table 2**, there was no significant difference in the initial Gn dose between the two groups. However, the total amount of Gn and number of Gn days in the fPPOS group were relatively longer than those in the antagonist group (*P* = 0.001 and *P* = 0.007, respectively). This finding was attributed to the slow development of small follicles and the delayed response to FSH in the fPPOS group. At the beginning, the E2, LH, and FSH levels of the fPPOS group were higher than those of the antagonist group (*P* = 0.000, *P* = 0.000, and *P* = 0.010, respectively). Elevated E2 levels suggested early growth of subsequent large follicles, while high levels of LH and FSH may have been associated with a poor response of subsequent small follicles to FSH.

**Table 2:** Data during controlled ovarian stimulation in the two groups.

GROUP	fPPOS (n=129)	GnRH antagonist (n=258)	t/Z/chi-square value	P
Initial Gn dose (IU)	225 (150-300)	225 (150-300)	-0.484	0.629
Gn dosage (IU)	2700 (1800-3287.5)	2100 (1650-2700)	-3.421	0.001
Gn usage days	10 (9-13)	10 (8-11)	-2.712	0.007
FSH at initial Gn (IU/L)	7.91 (6.25-9.41)	7.05 (6-8.47)	-2.560	0.010
LH at initial Gn (IU/L)	5.9 (4.59-8.92)	4.98 (3.75-6.38)	-3.915	0.000
E2 at initial Gn (pg/ml)	44.07 (30.24-76.34)	36 (26.94-45.77)	-3.868	0.000
P at initial Gn (ng/ml)	0.23 (0.11-0.47)	0.24 (0.12-0.38)	-.449	0.653
Proportion of large follicles (>=10mm) on the fifth day of Gn	14.15% (220/1555)	19.42% (646/3327)	20.161	0.000
E2 on the fifth day of Gn (pg/ml)	313.95 (128.05-681.78)	374.30 (224.10-610.10)	-1.851	0.064
LH on the fifth day of Gn (IU/L)	4.71 (2.79-7.48)	2.41 (1.69-3.81)	-6.797	0.000
P on the fifth day of Gn (ng/ml)	0.28 (0.15-0.52)	0.187 (0.098-0.32)	-2.983	0.003
LH on trigger day (IU/L)	3.2 (1.93-5.88)	2.96 (1.91-4.79)	-1.226	0.220
E2 on trigger day (pg/ml)	1725 (810.75-3315.5)	1873 (1111-3000)	-.106	0.916
P on trigger day (ng/ml)	0.58 (0.3-1.05)	0.64 (0.38-0.96)	-.355	0.722
Proportion of P>3ng/ml on trigger day	9.68% (12/124)	0.40% (1/252)	21.444	0.000
The proportion of follicles ≥ 14mm on the trigger day	6 (3-11)	6 (4-10)	-.922	0.357
Endometrial thickness on trigger day (mm)	9 (7-11)	10 (8-11)	-3.733	0.000

Gn, gonadotropinh; FSH, follicle stimulating hormone; LH, luteinizing hormone; E2, Estradiol; P, Progesterone. Statistical significance was reached at P < 0.05.

On the fifth day of Gn administration, the two groups showed distinct differences in the follicle size ratio and hormone levels. The proportion of large follicles (≥ 10 mm) in the antagonist group was significantly greater than that in the fPPOS group (*P* = 0.000), which also indicated that the growth of follicles in the antagonist group was more uniform. The P and LH levels in the fPPOS group were higher than those in the antagonist group (*P* = 0.000 and *P* = 0.003, respectively). Elevated levels of P and LH indicated that large follicles may have been at risk of premature ovulation.

On the trigger day, the E2, P, and LH levels did not differ significantly between the two groups. However, the proportion of P > 3 ng/ml on the trigger day in the fPPOS group was significantly higher than that in the antagonist group (*P* = 0.000). This is inevitable in the fPPOS protocol. This could also explain why the intimal thickness of the fPPOS group on the trigger day was slightly lower than that of the antagonist group (*P* = 0.000). The proportion of follicles ≥14 mm on the trigger day tended to be the same between the two groups, which also verified the consistency of our trigger timing control.

### Embryo Laboratory Data Between the Two Groups

The embryo laboratory data of the two groups are shown in **Table 3**. The number of retrieved oocytes in the antagonist group was slightly higher than that in the fPPOS group (*P* = 0.039). The number of total allocated metaphase II oocytes (IVF, ICSI, and PGT cycles) in the antagonist group was higher than that in the fPPOS group (*P* = 0.050). However, the number of allocated metaphase II oocytes (only for ICSI and PGT cycles) was equal in the two groups (*P* = 0.679). The number of allocated metaphase II oocytes (only for IVF cycles) in the antagonist group was higher than that in the fPPOS group (*P* = 0.019). The suboptimal oocyte yield in the antagonist group was higher than that in the fPPOS group (*P* = 0.022). The number of good-quality embryos in the antagonist group was slightly higher than that in the fPPOS group (*P* = 0.025). Since the basal antral follicle conditions between the two groups were matched, the aforementioned finding may have been related to the possibility of large follicle escape in the fPPOS group, while the antagonist group had uniform follicles and less large follicle escape. There were no significant differences in the rates of total fertilization, normal fertilization, good quality embryos, and blastocyst formation between the two groups, which supports the fact that the fPPOS protocol had no effect on oocyte maturation and subsequent embryonic development potential.

**Table 3:** Embryo laboratory data between the two groups.

GROUP	fPPOS (n=129)	GnRH antagonist (n=258)	t/Z/chi-square value	P
Number of Retrieved Oocytes	7 (3-13)	9 (5-14)	-2.061	0.039
Suboptimal Oocyte Yield	85.90% (1243/1447)	88.34% (2605/2949)	5.267	0.022
Number of Allocated Metaphase II Oocytes (total)	6 (2.5-11)	7 (4-11)	-1.964	0.05
Number of Allocated Metaphase II Oocytes (IVF)	5 (2-10.5)	7 (4-11)	-2.355	0.019
Number of Allocated Metaphase II Oocytes (ICSI+PGT)	6 (3.25-11)	7 (4-11)	-0.414	0.679
Total Fertilization Rate	79.03% (882/1116)	81.41% (1883/2313)	2.724	0.099
Normal Fertilization Rate	65.95% (736/1116)	67.75% (1567/2313)	1.103	0.294
Number of good Quality Embryos	3 (1-5)	3 (2-6)	-2.248	0.025
Rate of Good Quality Embryo	71.82% (520/724)	72.31% (1102/1524)	0.058	0.810
Rate of Blastocyst Formation	64.10% (350/546)	63.06% (565/896)	0.160	0.690

Statistical significance was reached at P < 0.05.

### Clinical Outcome Data

**Table 4** summarizes the basic data of the freeze-thaw transplant cycle and the main reproductive clinical outcomes of the two groups. At the deadline, 75 and 102 cases in the fPPOS and the antagonist groups, respectively, underwent FET. There was no significant difference in the age at transfer or the thickness of the endometrium on the day of transfer between the two groups. Moreover, there was no significant difference in the proportion of cleavage stage embryos and blastocyst stage embryos transferred between the two groups. Most importantly, we were most concerned about the non-significance of the difference in the rates of embryo implantation, clinical pregnancy, and early abortion between the two groups.

**Table 4:** Data of freeze-thaw transplantation cycle and reproductive outcome between the two groups.

GROUP		fPPOS (n=75)	GnRH antagonist (n=102)	t/Z/chi-square value	P
Number of Transplant cycles		75	102		
Age		33.89 ± 4.97	33.49 ± 5.21	0.519	0.605
Endometrial Thickness on Transplantation Day(mm)		9.16 ± 1.85	9.42 ± 2.13	-0.848	0.398
Type of Embryos Transferred	Cleavage Stage	61.33%	48.04%	3.072	0.080
	Blastocyst Stage	38.67%	51.96%		
Embryo Implantation Rate		46.83%	41.92%	0.702	0.402
Clinical Pregnancy Rate		61.33%	52.94%	1.238	0.266
Early Abortion Rate		13.04%	16.67%	0.256	0.613

Statistical significance was reached at P < 0.05.

### Part of the Data of Patients Undergoing PGT Assistance

To explore whether the fPPOS protocol initiated by this random transition affects the euploidy rate of embryos, we counted the embryo biopsies of patients who were included in the population for PGT assistance, as shown in **Table 5**. There were 17 and 12 cases in the fPPOS and the antagonist groups, respectively. The fPPOS group sent 72 embryos, 26 of which were euploid, while the antagonist group sent 52 embryos, 19 of which were euploid embryos. There was no significant difference in the euploidy rate and in the final average number of embryos transferred between the two groups. Because the number of patients in the PGT cycle is limited, we supplement other PGT cycle-related data (including the cause of PGT and the first frozen embryo transfer cycle after PGT) in the supplementary table (**Tables S1**, **S2**).

**Table 5:** Data of patients who performed PGT technology.

GROUP	fPPOS	GnRH antagonist	t/Z/chi-square value	P
Number of Ruploid Embryos/Number of Biopsy Embryos	36.11% (26/72)	36.54% (19/52)	0.002	0.961
Number of Transferable Embryos (PGT Diagnosis)	1.53	1.58	-0.068	0.947

PGT, preimplantation genetic testing. Statistical significance was reached at P < 0.05.

## Discussion

Our research found that flexible conversion to the fPPOS protocol could be considered if the follicles grow unevenly in the ovarian stimulation cycle of the antagonist protocol. In this case, pregnancy outcomes of the fPPOS protocol were comparable to those of the standard fixed antagonist protocol. Although the number of eggs obtained and the number of good quality embryos were slightly lower, the rates of embryo implantation, clinical pregnancy, and early abortion were comparable.

The human reproductive cycle is characterized by development of a single follicle and ovulation. Most follicles undergo three stages, namely follicle recruitment, selection, and dominance to maturity (25). Under physiological conditions, when FSH reaches the threshold, it begins to recruit follicles with a diameter of 2 to 5 mm (26). Its duration, characterized by the FSH window period, determines the number of follicles to be recruited and the formation of only one dominant follicle (27). In the late stage of follicle development, when the diameter of the follicle is approximately 9 mm, the follicle begins to differentiate. The dominant follicle continues to grow, while the remaining follicles become atretic (28). The lowest LH threshold level is necessary for follicular development. Follicle development stops when the LH level exceeds the upper limit of follicle development. The threshold and window period of FSH and LH play an important role in the recruitment, development, selection, and maturation of follicles. This causes follicles to grow unevenly during follicular development.

Animal model studies showed that progesterone inhibited the surge of GnRH/LH before ovulation through the hypothalamic progesterone receptor (29, 30). The exact mechanism by which progesterone inhibited LH secretion remained unclear. The stimulating effect of estradiol on the pulse frequency of GnRH may have been blocked by P (31). MPA suppressed the surge in LH. In addition, MPA had no effect on oocyte quality or pregnancy outcomes. Previous studies by our team showed that in patients with poor ovarian responders (POR) (25), the ovulation induction cycle was unified. Furthermore, the rate of ovulation induction in the luteal phase was even higher than that in the follicular phase, although our data sample size was limited.

Our research supports the concept that the combination of exogenous and endogenous P, which increased, had no effect on oocyte quality or pregnancy outcomes. As shown in **Table 5**, the euploidy rate of embryos in several patients undergoing PGT indicated no significant difference between the two groups. It also preliminarily supports the view that the fPPOS protocol in the case of uneven follicular growth did not affect the euploidy rate of embryos.

There were several advantages of switching to the fPPOS protocol. First, there was no need for daily injections. Only cheaper oral medications were needed. Although it was not possible to transfer fresh embryos, the cost of recycled FET indirectly compensated for the reduced cost of oral medications compared to injections. Second, in patients with uneven follicle growth, a timely and flexible transformation was achieved. This protocol was independent of the impact of subsequent elevated P levels on endometrial receptivity and effectively suppressed the LH peak, thus achieving clinical outcomes comparable to traditional antagonist regimens. Our research further suggested that the euploidy rate of embryos with the fPPOS protocol in patients undergoing PGT-assisted pregnancy was not affected. Third, the control of the starting dose of the antagonist protocol (whether flexible or fixed) was often difficult to grasp and unstable. The growth of follicles was prone to be uneven. An artificially low response after routine treatment was observed. Our research integrated the antagonist protocol and the fPPOS protocol, which provided additional information on the antagonist protocol and a new direction for future research. This remedied the poor response after the antagonist protocol was initiated. Fourth, the learning curve of the antagonist protocol also focused on the endometrial receptivity of antagonists in recent years. In the case of uneven follicle growth, it was easier to incorporate changes in the endometrial implant window. The conversion to the fPPOS protocol circumvented the concern regarding tolerance to a certain extent because such patients rejected the fresh cycle transfer.

However, flexible conversion of the PPOS protocol also had shortcomings. First, the protocol could not be implemented with a fresh cycle transfer, and the overall dosage and number of days used for Gn were relatively greater. However, this situation was also associated with poor response of early small follicles to FSH. Second, the large follicles grown in the first wave were discarded when the eggs were retrieved, while small follicles grown in the second wave were obtained. However, these findings further verified the existence and feasibility of the follicular wave theory.

There were advantages to this retrospective study. This was the first retrospective study exploring the fPPOS protocol in patients with uneven follicular growth. First, this study applied model matching to the fixed antagonist protocol. AFC and AMH predicted the effectiveness of ovulation stimulation to a certain extent. The effectiveness of ovulation stimulation was generally reflected in the number of eggs obtained and the maturity of the oocytes. The rate of blastocyst formation was an important indicator. This was also the source of data for the selection of matching indicators. Second, previous studies focused on egg donor cycles or POR patients, while our research focused on their own IVF/ICSI cycles. Furthermore, our research targeted normal responders.

This study also has several limitations. First, this was a retrospective study with some inherent limitations, although all efforts were made to control potential selection bias. Second, the definition of uneven follicle growth or poor Gn response was not unified. Instead, we used an empirical definition. In this experiment, our patient selection criteria were derived from our own definition. Third, we matched the patients using a fixed antagonist protocol with relatively consistent follicular homogeneity. Owing to the limitations of the retrospective analysis of data, we did not match the same antagonist patients with uneven follicular growth. We attempted to use the most classic and most fixed antagonist population model matching to verify the feasibility of our fPPOS protocol.

In conclusion, our research found that it was feasible to switch to the fPPOS protocol flexibly if the follicles grew unevenly in the ovarian stimulation cycle of the antagonist protocol. In this case, the fPPOS protocol pregnancy outcomes were comparable to those with the standard fixed antagonist protocol. Although the number of eggs obtained and the number of good quality embryos were slightly lower, the rates of embryo implantation, clinical pregnancy, and early abortion were comparable.

## Data Availability Statement

The original contributions presented in the study are included in the article/**Supplementary Material**. Further inquiries can be directed to the corresponding authors.

## Ethics Statement

Written informed consent was obtained from the individual(s) for the publication of any potentially identifiable images or data included in this article.

## Author Contributions

LS and MD was responsible for experiment conception, collection, and analysis of data. LH and FW was responsible for experiment design, experiment conception and manuscript writing. XZ and FL designed the work, provided technical guidance and final approved of manuscript. All authors read and approved the final manuscript.

## Funding

The whole study was supported by Natural Science Foundation of Guangdong Province (No. 2016A030313817), Science and Technology Program of Guangzhou, China (201704020217) and Science and Technology Program of Guangzhou, China (202102080432).

## Conflict of Interest

The authors declare that the research was conducted in the absence of any commercial or financial relationships that could be construed as a potential conflict of interest.

## Publisher’s Note

All claims expressed in this article are solely those of the authors and do not necessarily represent those of their affiliated organizations, or those of the publisher, the editors and the reviewers. Any product that may be evaluated in this article, or claim that may be made by its manufacturer, is not guaranteed or endorsed by the publisher.

## Supplementary Material

Supplementary Table 1 The indications of patients who performed PGT technology. PGT, preimplantation genetic testing.

Supplementary Table 2 Cycle data and clinical outcome of patients who performed PGT technology. PGT, preimplantation genetic testing.
