# Supplementary material for: Fixed Gonadotropin-Releasing Hormone Antagonist Protocol Versus Flexible Progestin-Primed Ovarian Stimulation Protocol in Patients With Asynchronous Follicular Development During Controlled Ovulation Stimulation: A Retrospective Study
Source: Front Endocrinol (Lausanne). 2021 Nov 18;12:690575. doi: 10.3389/fendo.2021.690575 (PMC8636937; doi:10.3389/fendo.2021.690575)
Supplement: Supplementary Table 1 — The indications of patients who performed PGT technology. PGT, preimplantation genetic testing. [file Table_1.doc]

Table S1 The indications of patients who performed PGT technology

|  |  | Number of Oocyte Retrieval cycles | | Number of Biopsy cycles | |
| --- | --- | --- | --- | --- | --- |
| PGT method | Genetic etiology | fPPOS | GnRH antagonist | fPPOS | GnRH antagonist |
| PGT-A | Adverse Pregnancy History | 5 | 1 | 3 | 1 |
|  | Repeated Implantation Failure | 3 | 2 | 1 | 0 |
|  | Recurrent Spontaneous Abortion | 13 | 9 | 7 | 5 |
|  | Elderly Patients | 2 | 3 | 1 | 1 |
| PGT-A Summary |  | 23 | 15 | 12 | 7 |
| PGT-M | α-Thalassaemia |  | 2 |  | 1 |
|  | β-Thalassaemia | 1 |  |  |  |
|  | Single-Gene Defects（DYNC2H1） |  | 1 |  | 1 |
|  | Single-Gene Defects (DMD) | 1 |  | 1 |  |
| PGT-M Summary |  | 2 | 3 | 1 | 2 |
| PGT-SR | pericentric inversion of chromosome |  | 1 |  | 0 |
|  | Robertson Translocation | 2 | 3 | 1 | 2 |
|  | Balanced Chromosome Translocation | 4 | 5 | 3 | 3 |
|  | Chromosomal Mumerical Abnormality |  | 2 |  | 1 |
| PGT-SR Summary |  | 6 | 11 | 4 | 6 |
| Summary |  | 31 | 29 | 17 | 15 |

PGT, preimplantation genetic testing.
