# Supplementary material for: Fixed Gonadotropin-Releasing Hormone Antagonist Protocol Versus Flexible Progestin-Primed Ovarian Stimulation Protocol in Patients With Asynchronous Follicular Development During Controlled Ovulation Stimulation: A Retrospective Study
Source: Front Endocrinol (Lausanne). 2021 Nov 18;12:690575. doi: 10.3389/fendo.2021.690575 (PMC8636937; doi:10.3389/fendo.2021.690575)
Supplement: Supplementary Table 2 — Cycle data and clinical outcome of patients who performed PGT technology. PGT, preimplantation genetic testing. [file Table_2.doc]

Table S2 Cycle data and clinical outcome of patients who performed PGT technology

| GROUP | fPPOS | GnRH antagonist |
| --- | --- | --- |
| Number of PGT Oocyte Retrieval cycles | 31 | 29 |
| Number of PGT Biopsy cycles | 17 | 15 |
| Number of PGT Embryo transfer cycles | 10 | 9 |
| Number of Clinical Pregnancy | 9 | 6 |
| Number of Early Abortion | 1 | 0 |
| Clinical Pregnancy Rate | 90.00% | 66.67% |
| Early Abortion Rate | 11.11% | 0.00% |

PGT, preimplantation genetic testing.
